# Supplementary material for: Structural basis of substrate recognition and translocation by human ABCA4
Source: Nat Commun. 2021 Jun 22;12:3853. doi: 10.1038/s41467-021-24194-6 (PMC8219669; doi:10.1038/s41467-021-24194-6)
Supplement: Supplementary file 1 — Supplementary Information [file 41467_2021_24194_MOESM1_ESM.pdf]

Supplementary Information

Structural basis of substrate recognition and translocation by human ABCA4

Tian Xie[1,2], Zike Zhang[1,2], Qi Fang[1], Bowen Du[1] and Xin Gong[1,*]

[1]Department of Biology, Southern University of Science and Technology, Shenzhen, Guangdong 518055, China.

[2]These authors contributed equally to this work.

[*]Corresponding author. Email: gongx@sustech.edu.cn

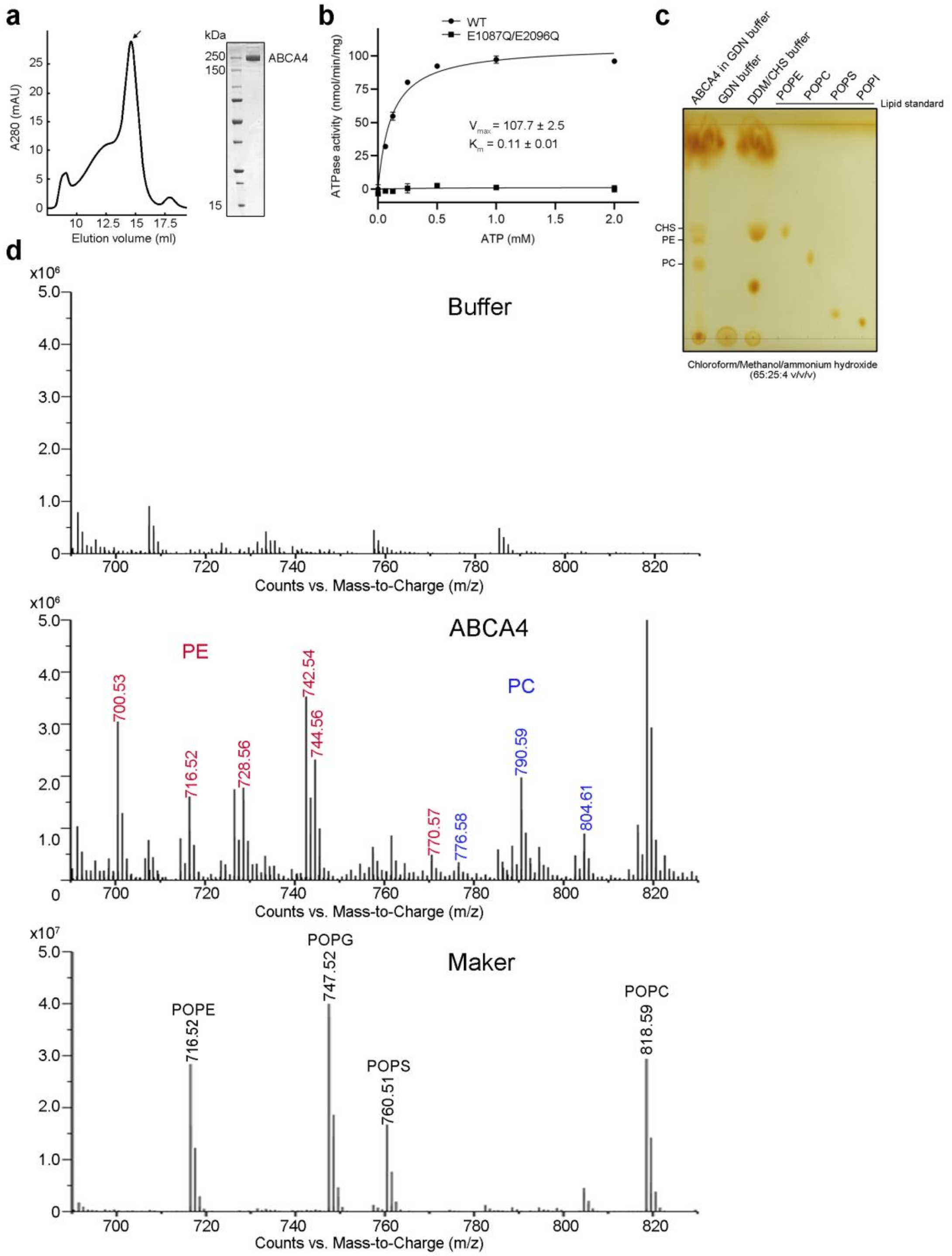

Supplementary Fig. 1 | Biochemical characterizations of human ABCA4. a, Size exclusion chromatography of human ABCA4. The peak fractions indicated by the arrow were pooled and concentrated for biochemical and structural studies. The purified protein was visualized by Coomassie-blue stained SDS-PAGE. The

SDS-PAGE is a representative of at least 10 independent experiments with similar results. **b,** Specific ATPase activity versus ATP concentration measured using wild-type (WT) ABCA4 protein or the catalytic mutant (E1087Q/E2096Q, $ABCA4_{QQ}$). Both constructs were purified in GDN detergent. Each data point is the average of three independent experiments, and error bars represent the s.d. **c,** Detection of endogenous lipids co-purified with ABCA4 by TLC. **d,** Mass spectrometry analysis of lipids co-purified with ABCA4. The upper and lower panels represent the mass spectra data for lipids purified from buffer (as a blank) and phospholipid standards, respectively. The middle panel shows the mass spectra of lipids co-purified with ABCA4. The ions at *m/z* 700.53, 716.52, 728.56, 742.54, 744.56, and 770.57 were identified as PE. The ions at *m/z* 776.58, 790.59, and 804.61 were identified as PC. All the ions were confirmed by tandem MS spectra. Source data for **a-c** are provided as a Source Data file.

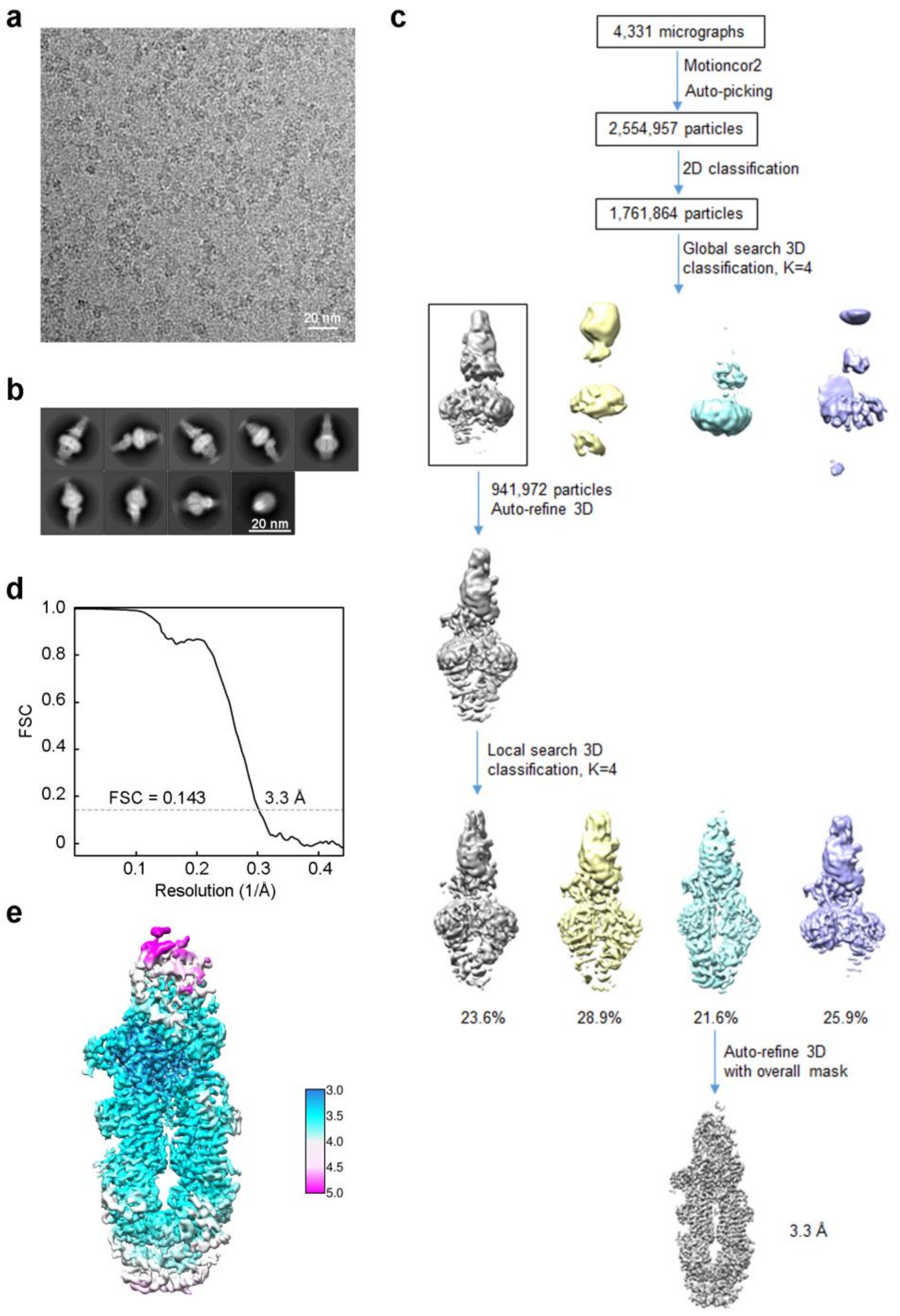

Supplementary Fig. 2 | Cryo-EM analysis of apo ABCA4. a, Representative cryo-EM micrograph. The micrograph is a representative of the 4,331 cryo-EM images. **b**, Representative 2D class averages. **c,** Flowchart for cryo-EM data processing. **d,** Gold-standard FSC curve for ABCA4 map generated using Relion 3.0. **e,** Local resolution map of ABCA4. The color code for resolutions, shown with the unit Å, is calculated using Relion 3.0.

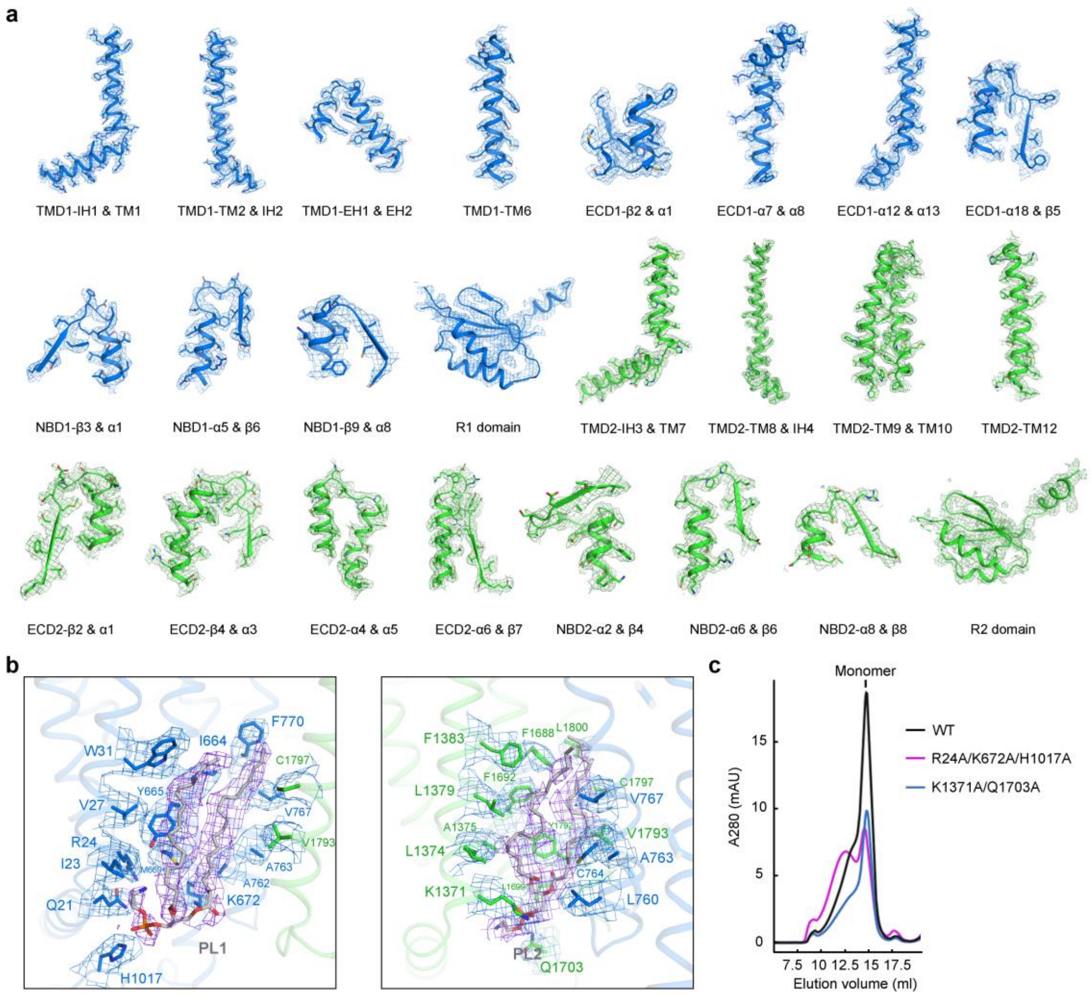

Supplementary Fig. 3 | EM maps for representative segments and phospholipid binding sites of apo ABCA4 and purification of ABCA4 mutants related to phospholipid binding. **a,** Electron density maps, contoured at 5 σ, for representative segments of apo ABCA4. **b**, Electron density maps, contoured at 4 σ, for phospholipid binding sites of apo ABCA4. **c,** The size-exclusion chromatography profiles of ABCA4 mutants related to phospholipid binding. The monomer peak fractions of ABCA4 mutants were collected for biochemical studies.

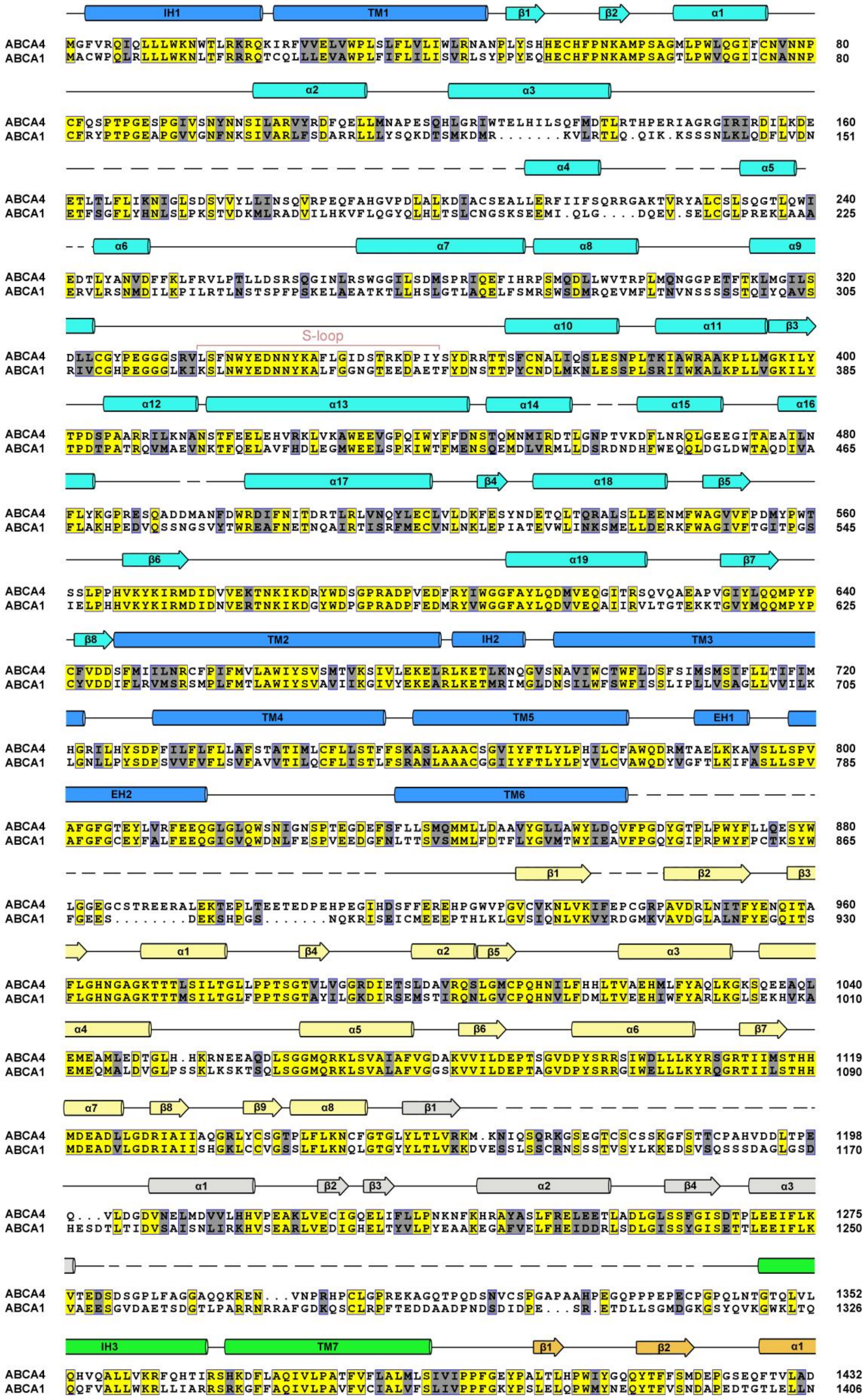

ABCA4
ABCA1
IH1
TM1
β1
β2
α1
α2
α3
α4
α5
α6
α7
α8
α9
S-loop
α10
α11
β3
α12
α13
α14
α15
α16
α17
β4
α18
β5
β6
α19
β7
β8
TM2
IH2
TM3
TM4
TM5
EH1
EH2
TM6
IH3
TM7

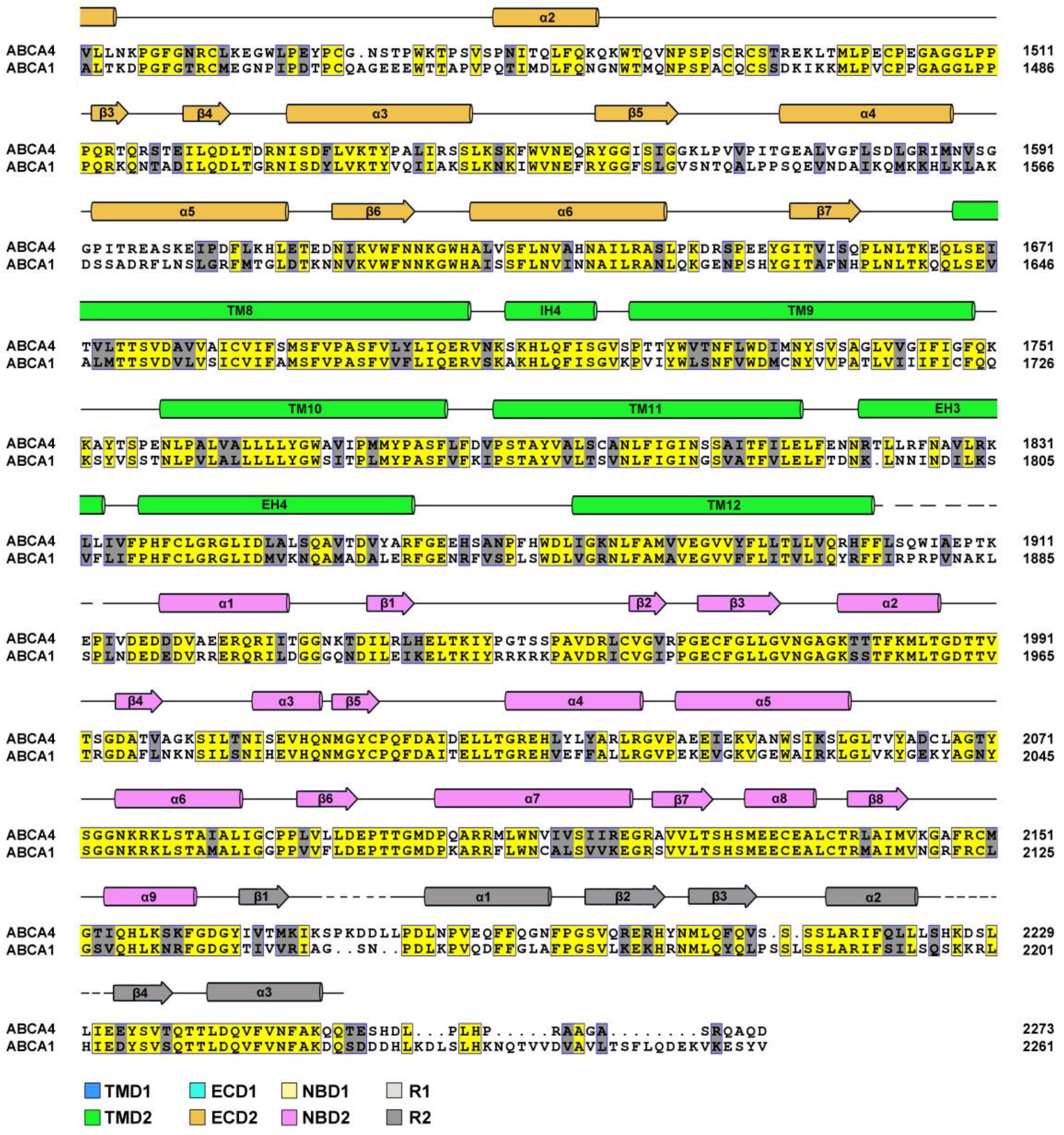

Supplementary Fig. 4 | Sequence alignment of human ABCA4 and ABCA1. Secondary structural elements of human ABCA4 are indicated above the sequence alignment. Dashed lines indicate protein portions that were not resolved in the cryo-EM map. Invariant and highly conserved amino acids are shaded yellow and gray, respectively. The secondary structural elements are colored based on the domains, with TMD1 and TMD2 colored marine and green; ECD1 and ECD2 colored cyan and orange; NBD1 and NBD2 colored yellow and magenta; R1 and R2 colored light gray and dark gray.

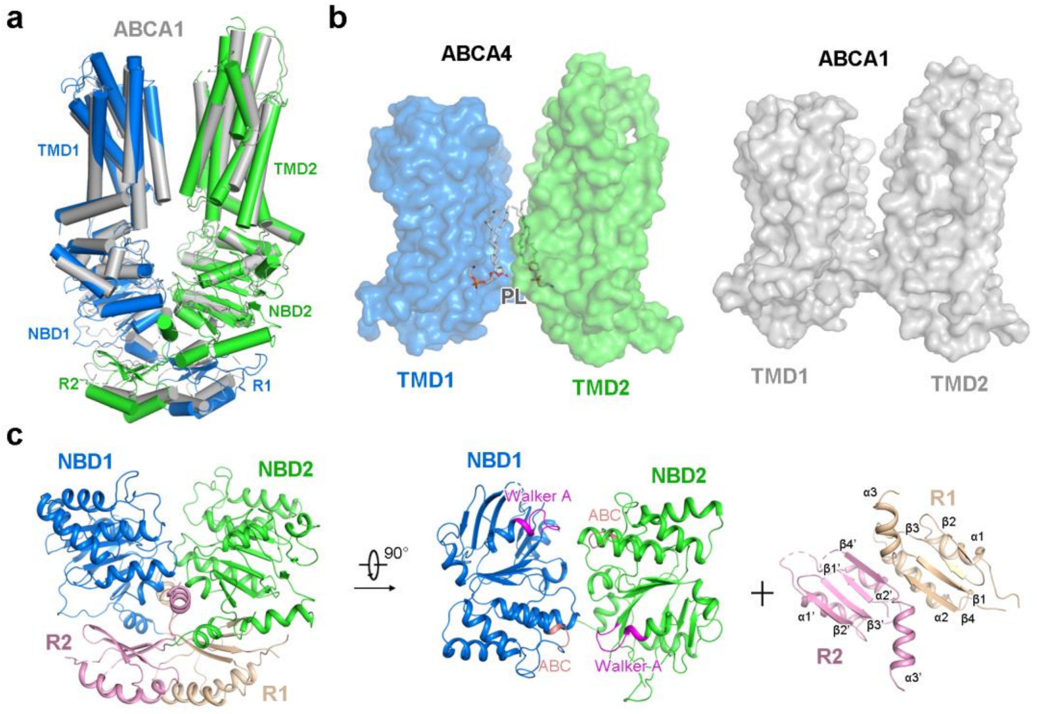

Supplementary Fig. 5 | Structural features of the TMDs, NBDs, and RDs of ABCA4. **a,** Superposition of ABCA4 and ABCA1 structures with ECDs omitted. ABCA1 structure is colored gray. TMD1, NBD1, and R1 of ABCA4 structure are colored marine. TMD2, NBD2, and R2 of ABCA4 structure are colored green. **b**, Surface representations of the TMDs from ABCA4 and ABCA1 structures. The phospholipids bound in the cytoplasmic leaflet of ABCA4 are shown as gray sticks. **c,** Structure of the NBDs and RDs of ABCA4. NBD1 and NBD2 are colored marine and green, respectively. R1 and R2 are colored wheat and pink, respectively. The Walker A motifs and ABC signature sequences are highlighted in magenta and light pink, respectively.

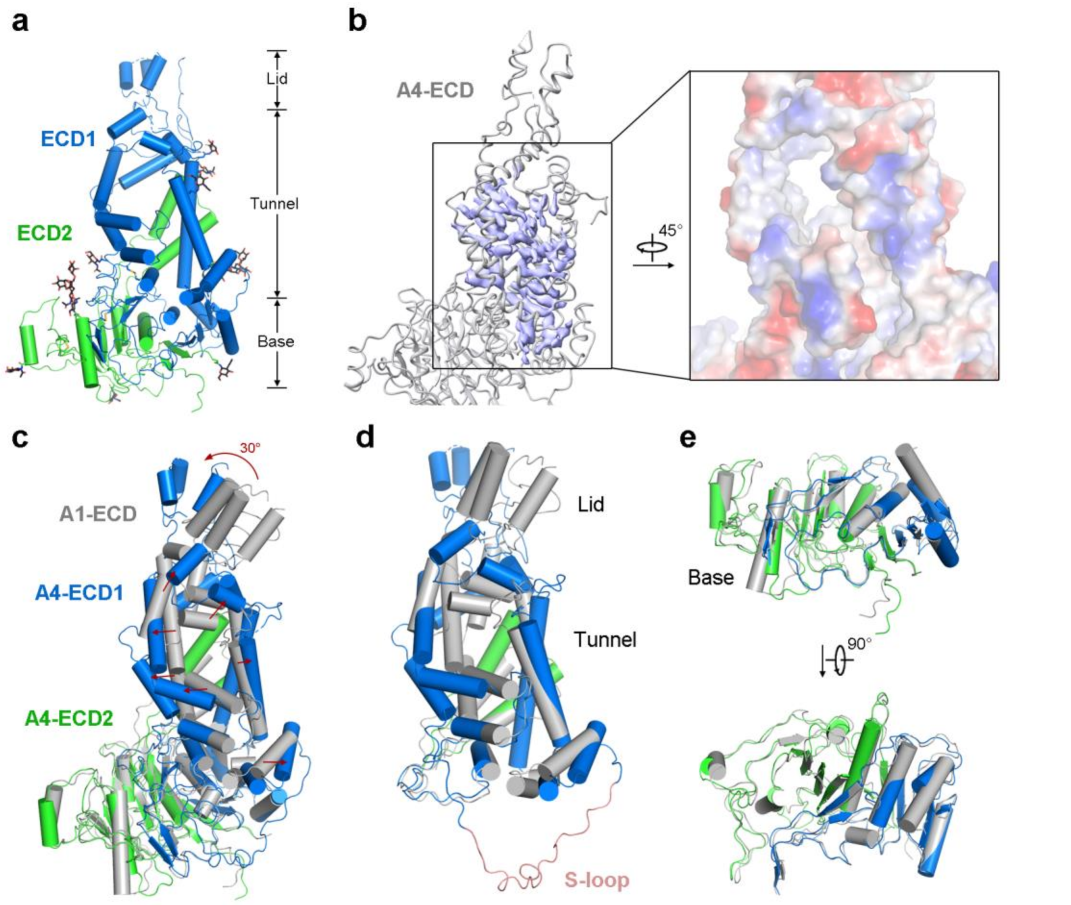

Supplementary Fig. 6 | Structural features of the ECD of ABCA4. **a,** The ECD of ABCA4 exhibits a three-layer structural organization, consisting of lid, tunnel, and base. **b**, A hydrophobic tunnel within the ECD of ABCA4 is filled with lipid-like densities. The lipid-like densities in the left panel are displayed in purple. The ECD tunnel in the right panel is shown as surface electrostatic potential. **c,** Superposition of the ECD structures from ABCA4 and ABCA1. The ECD from ABCA1 (A1-ECD) is colored gray. ECD1 and ECD2 of ABCA4 are colored marine and green, respectively. **d,** Structural differences in the tunnel and lid regions from A4-ECD and A1-ECD. The S-loop from A4-ECD tunnel is colored light pink. The corresponding region from A1-ECD tunnel was missing due to structural flexibility. **e,** Close-up views of the superimposed ECD bases.

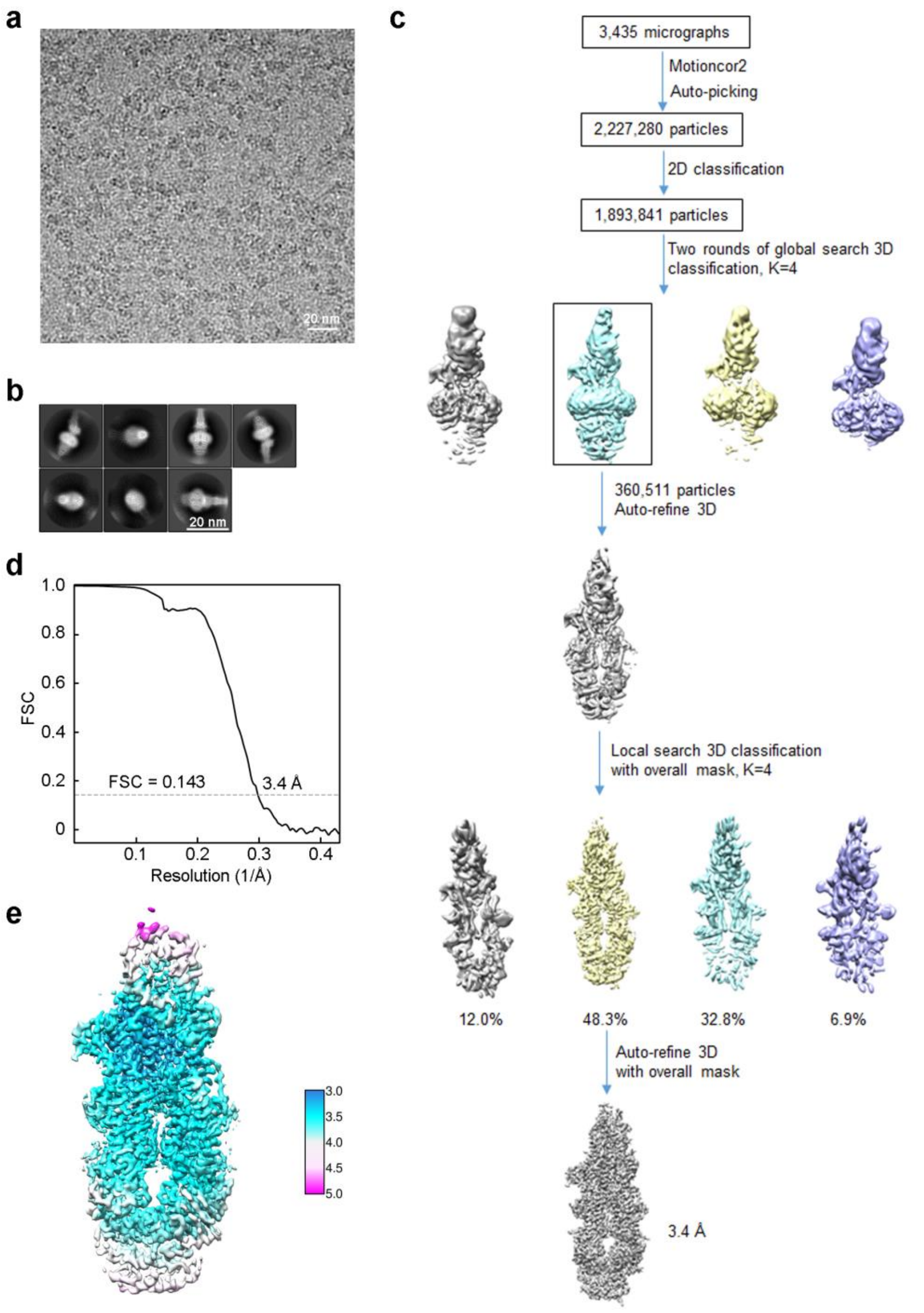

Supplementary Fig. 7 | Cryo-EM analysis of NRPE-bound ABCA4. a, Representative cryo-EM micrograph. The micrograph is a representative of the 3,435 cryo-EM images. **b**, Representative 2D class averages. **c,** Flowchart for cryo-EM data processing. **d,** Gold-standard FSC curve for NRPE-bound ABCA4 map. **e,** Local resolution map of NRPE-bound ABCA4.

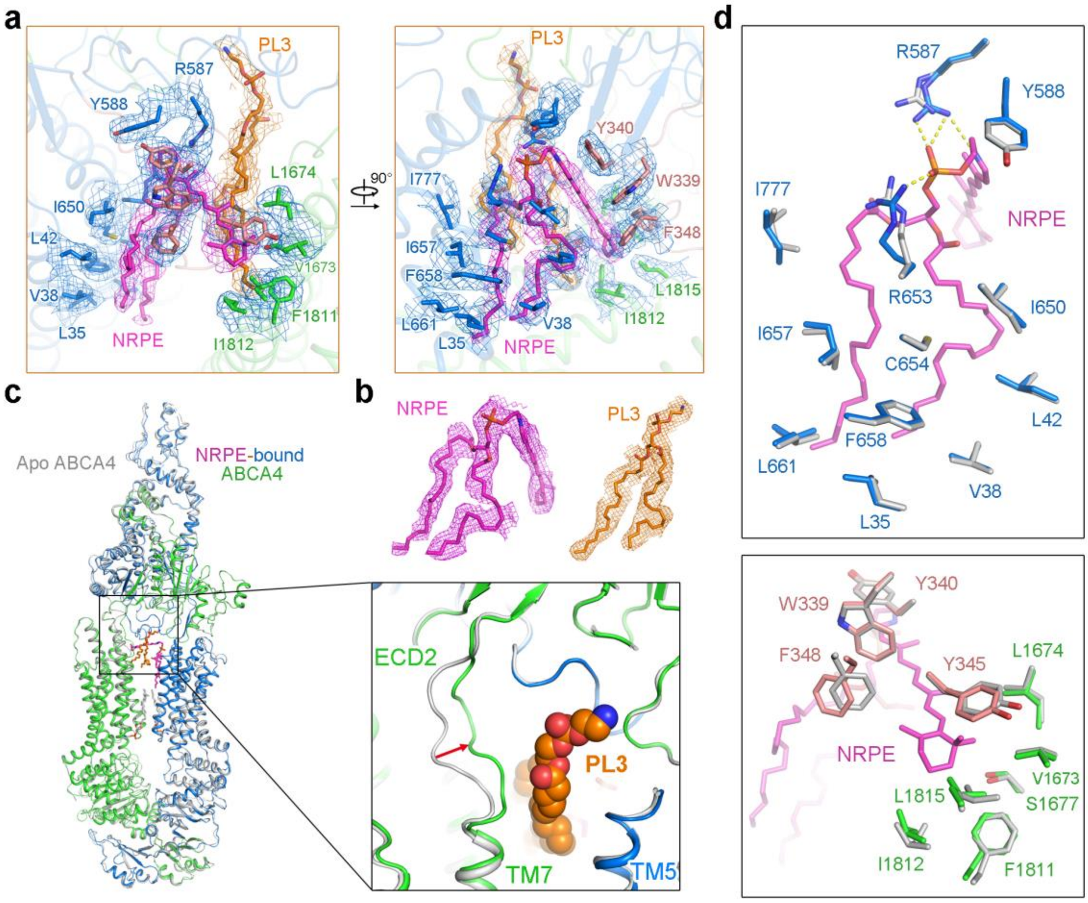

Supplementary Fig. 8 | EM maps for NRPE binding site and a small conformational change of ABCA4 upon NRPE binding. **a,** EM maps for the NRPE binding site contoured at 4 σ. **b**, EM maps for NRPE and PL3 bound in the luminal membrane leaflet contoured at 4 σ. **c,** A small conformational change of the connecting loop between TM7 and ECD2 upon NRPE and PL3 binding. Apo ABCA4 is colored gray. NRPE-bound ABCA4 is colored marine and green. PL3 is shown as orange spheres. **d,** Comparison of the NRPE-binding pocket between apo and NRPE-bound ABCA4 structures. The residues of apo ABCA4 is colored gray. The residues of NRPE-bound ABCA4 are colored marine, green, and light pink. NRPE is shown in magenta sticks.

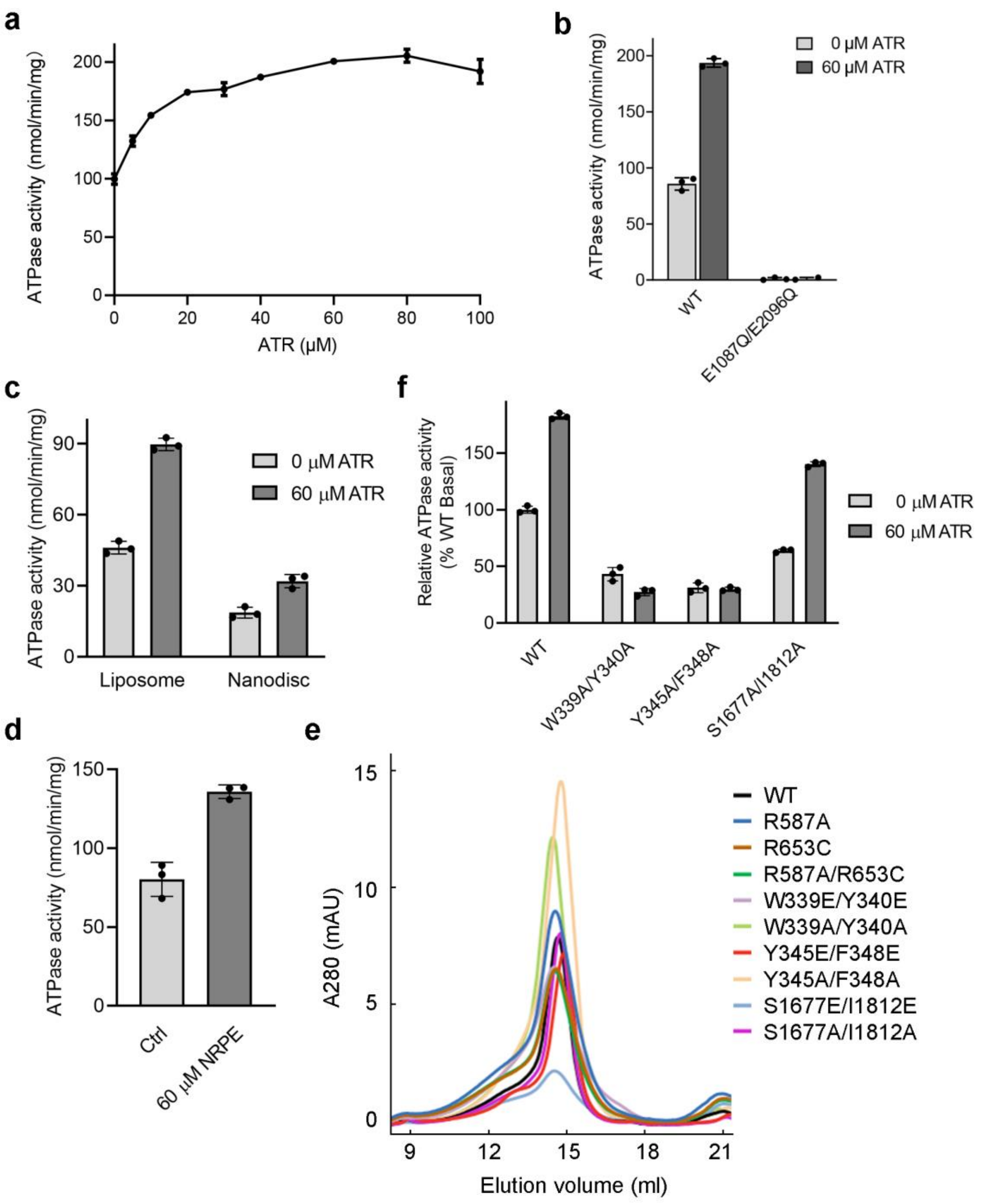

Supplementary Fig. 9 | ATR stimulation of the ATPase activity of ABCA4 and purification of ABCA4 mutants related to NRPE binding. **a**, Specific ATPase activity of WT ABCA4 (purified in CHAPS/lipid mixture) as a function of ATR concentration. **b**, Specific ATPase activity of WT ABCA4 or the catalytic mutant (E1087Q/E2096Q) in the absence or presence of 60 μM ATR. Both constructs were purified in CHAPS/lipid mixture. **c**, Specific ATPase activity of WT ABCA4

(reconstituted in liposome or nanodisc) in the absence or presence of 60 μM ATR. **d**, Specific ATPase activity of WT ABCA4 (purified in GDN) in the absence or presence of 60 μM NRPE. **e**, The size-exclusion chromatography profiles of ABCA4 mutants related to NRPE binding. **f**, ATR-stimulated ATPase activity measurement for the ABCA4 variants with mild alanine substitutions of key residues involved in NRPE coordination. The ATPase activity was normalized relative to the basal ATPase activity of the WT ABCA4. For all curve and dot-plot graphs, each data point is the average of three independent experiments, and error bars represent the s.d. Source data for **a-d** and **f** are provided as a Source Data file.

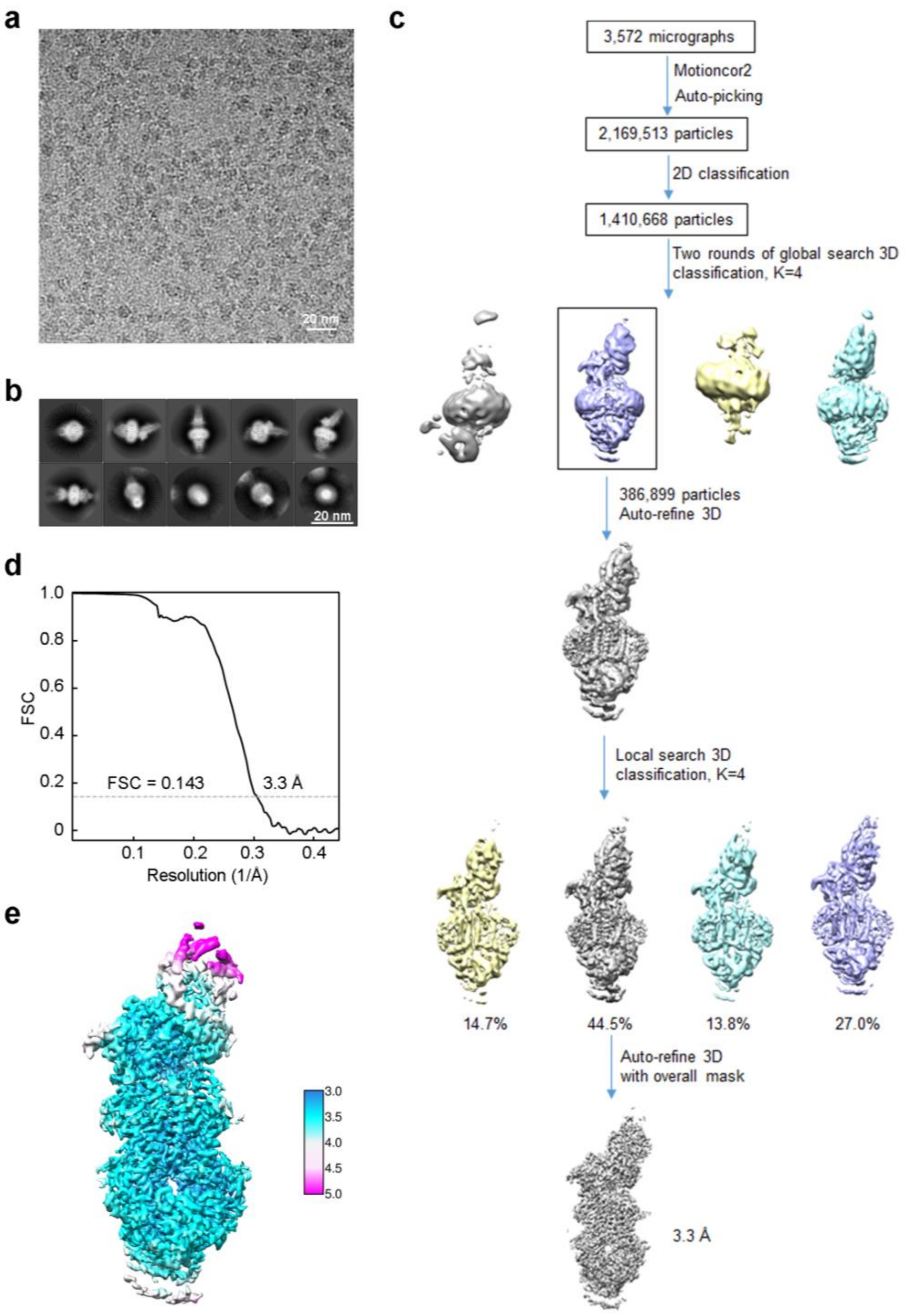

Supplementary Fig. 10 | Cryo-EM analysis of ATP-bound ABCA4. a, Representative cryo-EM micrograph. The micrograph is a representative of the 3,572 cryo-EM images. **b**, Representative 2D class averages. **c,** Flowchart for cryo-EM data processing. **d,** Gold-standard FSC curve for ATP-bound ABCA4 map. **e,** Local resolution map of ATP-bound ABCA4.

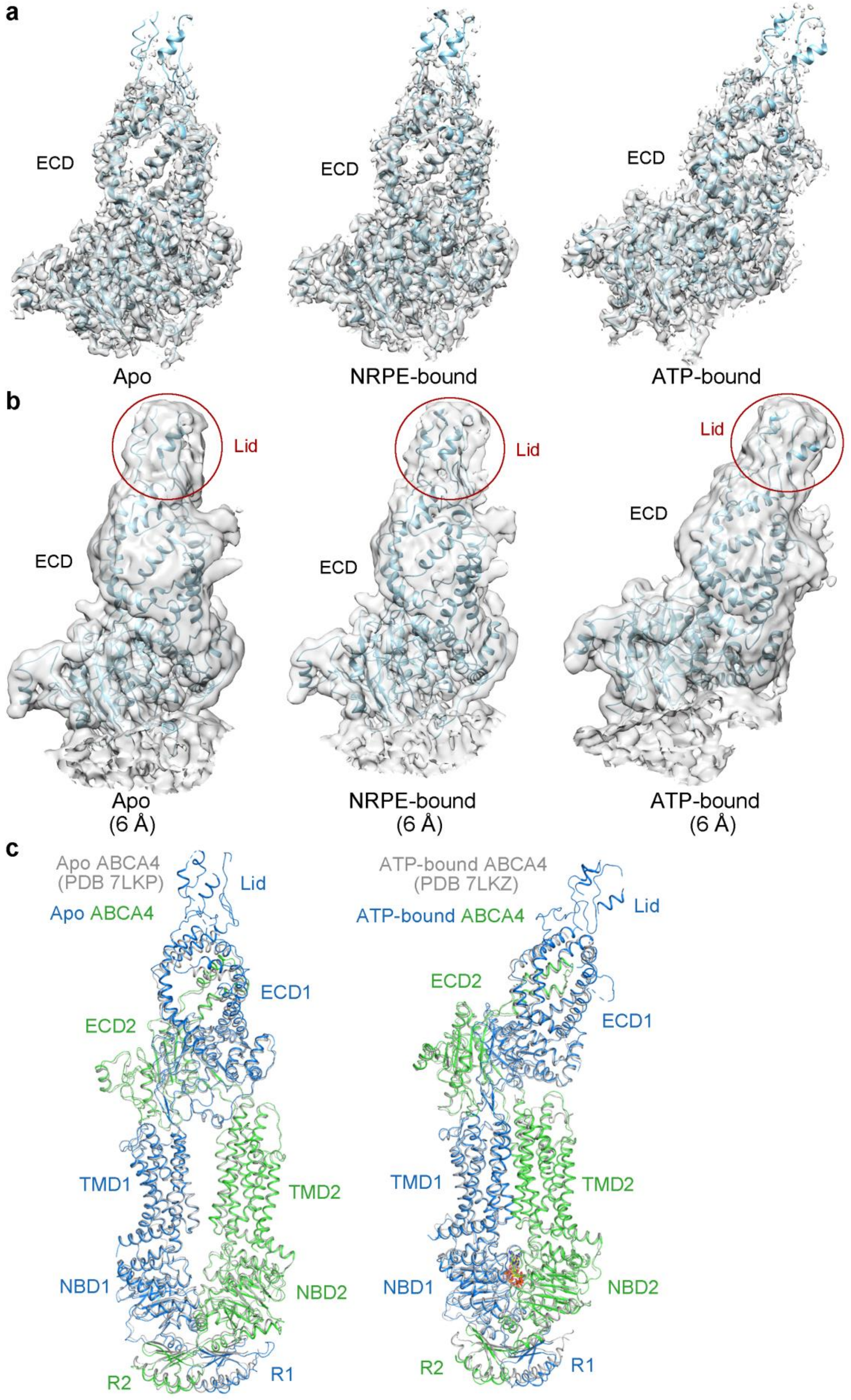
a
ECD
ECD
ECD
Apo
NRPE-bound
ATP-bound
b
Lid
Lid
Lid
ECD
ECD
ECD
Apo
(6 Å)
NRPE-bound
(6 Å)
ATP-bound
(6 Å)
c
Apo ABCA4
(PDB 7LKP)
Apo ABCA4
Lid
ECD1
ECD2
TMD1
TMD2
NBD1
NBD2
R2
R1
ATP-bound ABCA4
(PDB 7LKZ)
ATP-bound ABCA4
Lid
ECD2
ECD1
TMD1
TMD2
NBD1
NBD2
R2
R1

Supplementary Fig. 11 | Cryo-EM maps of ECDs and structural comparison of the ABCA4 structures. **a,** Cryo-EM maps of ECDs from the apo (left panel), NRPE-bound (middle panel) and ATP-bound (right panel) ABCA4 structures. The ECDs were presented in the same angle as that in Fig. 5d. **b**, Cryo-EM maps of ECDs low-pass filtered to 6 Å. The maps were presented in the same views as those in **a**. The EM densities for the lid region were indicated by red circles. **c**, Structural comparison of the ABCA4 structures. The published ABCA4 structures are colored gray (apo ABCA4: PDB 7LKP; ATP-bound ABCA4: PDB 7LKZ). The ABCA4 structures determined by us are colored marine and green.

Supplementary Table 1 | Cryo-EM data collection, refinement and validation statistics

	Apo ABCA4 (EMD-31000, PDB 7E7I)	NRPE-bound ABCA4 (EMD-31001, PDB 7E7O)	ATP-bound ABCA4 (EMD-31002 PDB 7E7Q)
**Data collection and processing**			
Magnification	130,000	130,000	130,000
Voltage (kV)	300	300	300
Electron exposure (e^{-}/Å^2)	50	50	50
Defocus range (μm)	-2.0 to -1.0	-2.0 to -1.0	-2.0 to -1.0
Pixel size (Å)	1.08	1.08	1.08
Symmetry imposed	C1	C1	C1
Initial particle images (no.)	2,554,957	2,227,280	2,169,513
Final particle images (no.)	205,597	184,628	173,278
Map resolution (Å)	3.3 Å	3.4 Å	3.3 Å
FSC threshold	0.143	0.143	0.143
Map resolution range (Å)	3.0-5.0 Å	3.0-5.0 Å	3.0-5.0 Å
**Refinement**			
Initial model used (PDB code)	5XJY		
Model resolution (Å)	3.3 Å	3.4 Å	3.3 Å
FSC threshold	0.143	0.143	0.143
Map sharpening *B* factor (Å^2)	-119	-114	-114
Model composition			
Nonhydrogen atoms	15,703	15,820	15,203
Protein residues	2,007	2,006	1,966
Ligands	19	21	21
B factors (Å^2)			
Protein	55.71	61.35	54.44
Ligand	63.93	73.81	82.16
R.m.s. deviations			
Bond lengths (Å)	0.008	0.009	0.010
Bond angles (°)	0.968	0.970	1.002
**Validation**			
MolProbity score	2.20	2.13	2.04
Clashscore	10.04	9.16	8.40
Poor rotamers (%)	0.00	0.00	0.00
Ramachandran plot			
Favored (%)	83.91	86.18	88.39
Allowed (%)	15.78	13.57	11.20
Disallowed (%)	0.30	0.25	0.41

Supplementary Table 2 | Primers used in the study

**Primer**	**Sequence (5'-3')**
ABCA4-pCAG-1-NotI-F	GAGGTACCATGGCGAGCGGCCGCATGGGTTTCGTGCGCCAGAT
ABCA4-pCAG-2273-XhoI-R	AACCTCATCAGAGCCCTCGAGATCTTGAGCTTGGCGAGATG
ABCA4-E1087Q-F	GTGGTGATTCTGGACCAGCCCACCTCTGGTGTG
ABCA4-E1087Q-R	CACACCAGAGGTGGGCTGGTCCAGAATCACCAC
ABCA4-E2096Q-F	CTGGTGCTGCTGGATCAGCCTACCACCGGTATG
ABCA4-E2096Q-R	CATACCGGTGGTAGGCTGATCCAGCAGCACCAG
ABCA4-R24A-F	AAGCGTCAAAAGATCGCCTTCGTGGTGGAATTG
ABCA4-R24A-R	CAATTCCACCACGAAGGCGATCTTTTGACGCTT
ABCA4-K672A-F	GTGAGCATGACCGTGGCGTCCATCGTGCTGGAG
ABCA4-K672A-R	CTCCAGCACGATGGACGCCACGGTCATGCTCAC
ABCA4-H1017A-F	AACATCCTGTTCCACGCCCTGACTGTGGCTGAG
ABCA4-H1017A-R	CTCAGCCACAGTCAGGGCGTGGAACAGGATGTT
ABCA4-K1371A-F	ACCATCCGCAGCCACGCAGACTTCCTGGCCCAG
ABCA4-K1371A-R	CTGGGCCAGGAAGTCTGCGTGGCTGCGGATGGT
ABCA4-Q1703A-F	GTGCTGTACCTGATCGCGGAGCGCGTGAACAAG
ABCA4-Q1703A-R	CTTGTTCACGCGCTCCGCGATCAGGTACAGCAC
ABCA4-R587A-F	AACAAGATCAAGGACGCATACTGGGATTCTGGA
ABCA4-R587A-R	TCCAGAATCCCAGTATGCGTCCTTGATCTTGTT
ABCA4-R653C-F	ATGATCATCCTGAACTGCTGCTTCCCTATCTTC
ABCA4-R653C-R	GAAGATAGGGAAGCAGCAGTTCAGGATGATCAT
ABCA4-W339E/Y340E-F	GTGCTGTCCTTCAACGAGGAAGAGGACAACAACTAC
ABCA4-W339E/Y340E-R	GTAGTTGTTGTCCTCTTCCTCGTTGAAGGACAGCAC
ABCA4-Y345E/F348E-F	TACGAGGACAACAACGAAAAGGCCGAACTGGGTATTGACTCC
ABCA4-Y345E/F348E-R	GGAGTCAATACCCAGTTCGGCCTTTTCGTTGTTGTCCTCGTA
ABCA4-S1677E-F	ACTGTGCTGACCACCGAAGTTGATGCTGTGGTG
ABCA4-S1677E-R	CACCACAGCATCAACTTCGGTGGTCAGCACAGT
ABCA4-I1812E-F	AGCGCTATCACCTTCGAACTGGAGCTGTTCGAG
ABCA4-I1812E-R	CTCGAACAGCTCCAGTTCGAAGGTGATAGCGCT
ABCA4-W339A/Y340A-F	GTGCTGTCCTTCAACGCAGCCGAGGACAACAACTAC
ABCA4-W339A/Y340A-R	GTAGTTGTTGTCCTCGGCTGCGTTGAAGGACAGCAC
ABCA4-Y345A/F348A-F	TACGAGGACAACAACGCAAAGGCCGCTCTGGGTATTGACTCC
ABCA4-Y345A/F348A-R	GGAGTCAATACCCAGAGCGGCCTTTGCGTTGTTGTCCTCGTA
ABCA4-S1677A-F	ACTGTGCTGACCACCGCCGTTGATGCTGTGGTG
ABCA4-S1677A-R	CACCACAGCATCAACGGCGGTGGTCAGCACAGT
ABCA4-I1812A-F	AGCGCTATCACCTTCGCCCTGGAGCTGTTCGAG
ABCA4-I1812A-R	CTCGAACAGCTCCAGGGCGAAGGTGATAGCGCT
